# Report of the Meeting of the Western Dental Society, at Chicago, Ill., in the International Hall, July 30th, 1856

**Published:** 1856-10

**Authors:** 


### REPORT OF THE MEETING OF THE WESTERN DENTAL SOCIETY, AT CHICAGO, ILL., IN THE INTERNATIONAL HALL, JULY 30th, 1856.

*Present.*

| | |
|---|---|
| Dr. Allport, Chicago, Ill, | Dr. A. M. Kelsey, Geneva, Ill. |
| " J. C. Quinlan, Chicago, Ill. | " D. W. Perkins, Rome, N. Y. |
| " W. H. Kennicott, " | " H. R. Smith, Terre Haute, Ind. |
| " Kennicott, " | " C. P. Fitch, Milwaukee. |
| " E. Honsinger, " | " Hanson, " |
| " T. P. Abell, " | " I. P. Norman, Bockford, Ill. |
| " E A. Bogue, " | " Anderson, Hannibal, Mo. |
| " J. S. Clark, New Orleans, | " Solyman Brown, New York. |
| " J. S. Knapp, " | " Wm. Smith, Ottawa, Illinois. |
| " C. W. Spalding, St. Louis, | " A. T. Metcalf, Kalamazoo, Mich. |
| " A. Blake, " | " Mansfield, Niles, Mich. |
| " Henry Barron, " | " E. J. E. Carpenter, Joliet. |
| " S. Dunham, " | " C. J. Reynolds, Dixon, Ill. |
| " H. J. B. McKellops, " | " A. Gibbs, Chicago, Ill. |

Dr. H. N. Lewis, Quincy, Ill.

In the absence of the President, Dr. E. Hale, of St. Louis, Dr. Allport, one of the Vice Presidents, was called to the chair.

Dr. Allport said that, in the unexpected absence of the President, he should detain the meeting with but a very few remarks.

A few years had effected a remarkable change in the Dental profession. Formerly a knowledge of its more elevated practice was confined to the larger cities. Now the science of Dentistry was extended over the whole country, north, south, east, and west. This was the normal result of a generous liberality on the part of a few eminent practitioners, who had made known the principles and practice of Dentistry to their professional brethren, as well as to the students in the art, both privately and by means of periodicals and colleges, established expressly for the purpose, in various parts of the country. The names of Parmly, Harris, Maynard, Taylor, Townsend, Dwinelle, Solyman Brown, Dunning, Westcott, Clark, and others, would readily occur in this connection.

In pursuance of the same object, and in imitation of these noble examples, we organized this Society last April, in the Great West, and have now met for mutual instruction and improvement.

It was then, on motion, resolved, that Dr. Solyman Brown, of N. Y., Drs. Clark and Knapp, of N. O., Dr. Perkins, of Rome, N. Y., and Dr. Smith, of Terre Haute, Ind. (one of the Professors of the Ohio College of Dental Surgery), be invited to participate in the business of the present meeting.

After reading and approving the minutes of the last meeting, a committee consisting of Drs. Blake, Dunham, and Clark, reported as the special business of the session the following subjects for discussion, viz: The Extraction of the Deciduous Teeth of Children; the Plugging of the Fangs of the Teeth; The Use of Gutta Percha as the Basis of Artificial Teeth; and Crystal Gold.

This judicious selection of subjects was approved by the meeting.

Dr. Clark on being called upon opened the discussion on Deciduous Teeth. He said he deemed the subject one of pre-eminent importance, and if there was a point of practice that brought the careful operator the most cause for anxiety as to the adoption of correct practice, and of regret for popular mistakes and consequent mal-practice, it was the treatment of deciduous teeth.

These teeth had their use, and it was an important one, not only as furnishing temporary means of mastication, but in the production of a second permanent set; and if in any way they were wrested from that service, it must be considered as a misfortune. The first set, or deciduous teeth, were designed undoubtedly to answer the purpose of mastication, and to retain that office until the second teeth, developing under them, caused the loss of the deciduous fang by the process of absorption. Their retention, then, to that point, was a matter of importance.

Another point, and a material one, in this connection, he would mention. Immediately after the production of the deciduous teeth (twenty in number), came four protectors, in the shape of four permanent molars, one on each side above and below.

Their office was, by antagonizing, to protect the deciduous teeth in their coming days of weakness, and also to serve as pillars, holding the jaws in correct articulation, while the first are lost and the second set are developed.

Extract one of these (which mothers and nurses will always pronounce first, or deciduous teeth), and the jaw will tilt over until it meets with an antagonizing point. It may be a right articulation, but it is very apt to be a wrong one, and the cause of many lamentable cases of irregularity.

We have, then, clearly the indication for the preservation of these teeth and the deciduous ones, until they have performed their several functions.

He condemned the wholesale extracting of these teeth to order.

He did not extract one for ten brought to him for that purpose.

Dr. Kennicott inquired of Dr. Clark, what had been his successes in stopping deciduous teeth.

Dr. Clark responded, that he had never courted success in that line. He called it only *stuffing*.

Dr. Perkins remarked, that the development of the human physical system was harmonious throughout, and simultaneous in all its parts. He described the development of the two successive sets of human teeth. In treating the teeth of children he always regards primarily the welfare of the adult teeth which succeed them. The teeth of children are now developed much earlier in this country than in former years, the probable result of the different habits of society at successive periods.

Dr. Knapp inquired of Dr. Perkins, whether he would extract deciduous teeth, when they were decayed to the nerve; and was answered that it would depend somewhat on circumstances.

Dr. Knapp remarked, that children should be taught to exercise their teeth in the mastication of hard food, which is necessary to their healthy development.

Dr. Clark remarked, that Dr. Knapp had been trying the experiment, for four or five years, of brushing his children's teeth morning and evening, with his own hands, not being willing to entrust the experiment to nurses, and that his success in preserving them seemed thus far very perfect.

Dr. Perkins does not allow his children to use vinegar; in consequence of the deleterious effects of all acids, except those of the milder forms, such as found in fruits. He does this by way of experiment, as he thinks vinegar injures the tender enamel of children's teeth.

Dr. Dunham inquired whether there is any particular advantage in filling children's teeth.

Dr. Clark replied, that he used all means, such as cleanliness, treatment in case of pain, and stuffing or filling, to ensure their retention to as near the point of indication for removal as possible.

Dr. Allport stated, that he had been in the habit of doing so, when it seemed proper. When practicable he files instead of filling. He thinks that the subject of keeping children's teeth clean is of very great moment; on which point dentists ought to take great pains to instruct the public. Adjourned till 2 P. M.

AFTERNOON SESSION.

*The subject of "Children's Teeth" was resumed.*

Dr. Clark spoke at some length of the action of acids on the teeth, acids in food, acids in the secretions, and acids generated by foul deposits between and around the teeth.

He did not know, however, how far this crusade against acids should be carried, and had not, like Dr. Perkins, banished vinegar from his table.

He did not know that many of our acid fruits, or even vinegar, might not be only admissible, but really beneficial to the teeth in a healthy state. We ought to take into account physiological facts, as well as facts in chemistry.

To say that with the well known affinity of acids for lime, that the teeth are composed of some 80 per cent. lime, is rather alarming to the users of acetic or malic acid; but when we consider, physiologically, it is another matter.

Dr. Perkins believed that the luxuries of the present age, in this country, are destroying teeth with alarming rapidity, by vitriolizing the general constitution, as well as by direct action on the teeth.

Dr. Quinlan thinks that the experiments already made show clearly that acid substances injure the teeth.

Dr. Fitch thinks the great question is, what acids the food develops, and what action they have upon the teeth; and believing that the subject had been sufficiently discussed for the present, he suggested that the second question, "Fang Filling," be taken up for the afternoon discussion.

*Fang Filling.*

Dr. Clark being called on, said—

He had practiced the extirpating the nerve or pulp, and filling the canal occupied by it, for some nine or ten years, and that his present confidence in the operation is greater than any preceding expectations.

The history of this discovery was somewhat obscure. All he could say was, that it was performed many years previous to his attempts. The profession knew how far Dr. Koeker carried his operations in this direction, and it is certain that Drs. Maynard, Harwood, Badger, and Hudson practiced it years before any of us.

His method of operating he had long since given to the profession, which was to draw the temper from a fine broach, and to cut "fish-hook" barbs on one side, say two rows on two of the broach edges, with a sharp knife, leaving the other side perfectly smooth.

With the smooth side next the wall of the canal, he thrusts the instrument to the apex foramen; then carefully rotating and withdrawing the instrument, the nerve or pulp is caught on the barbs, and the pulp thus removed.

The operation of filling is the most simple part of it. Several broaches drawn to a blue, are loaded with strips of foil

(say one-eighth of an inch wide) from the point of the broach to as near the shape of the canal as possible.

One of these is inserted to the apex, the broach withdrawn from its cylinder of gold. A small smooth instrument is then thrust up by its side, and another loaded broach, as before, and so on until it is full. This method has one advantage. Should ulceration be threatened, and treatment be necessarily resumed, this filling can be easily removed by the tweezers.

If the pulp is entirely removed, or disease thoroughly eradicated, and this canal faithfully filled, we may hope for favorable results; but we should not promise our patients too much. Fang filling, from the very nature of the operation, must be somewhat uncertain. In fact, like all surgical operations, the operator is not the one to promise inevitably a cure.

We do not cure disease in any form. We merely remove obstructions and supply deficiencies, and nature does the rest.

Dr. Knapp said: The removal of the pulp of the fang is sometimes difficult; also the filling of the fang, especially of the molars. Creosote is the best agent for cleaning out the *pus* from the cavity.

Dr. Anderson uses creosote and arsenic to cleanse a nerve cavity and prepare it for filling. These substances are introduced on cotton by means of a broach.

Dr. Kennicott frequently extracts the nerve by the use of a piece of hickory, and sometimes stops the cavity with a well fitted hickory plug.

Dr. McKellops uses nitrate of silver, 60 grs. to the oz., to clear out the fang, and employs an instrument manufactured by the New York Teeth Manufacturing Company, which is excellent for the purpose.

Dr. Perkins uses the chloride of soda to inject the fangs of teeth. Sometimes the fang has an ulcer when there is no other disease in the tooth.

Dr. Clark treats ulcerated fangs by thrusting creosote up the orifice on cotton, and leaving it there from time to time for some days.

Dr. Dunham did not think favorably of the hickory plug, spoken of by Dr. Kennicott (in explanation of a case of his alluded to).

Dr. Clark stated that in cases where the fang of a molar with a lateral decay to the nerve, required to be stopped with gold, he was in the habit of drilling a hole through the grinding surface of the enamel, in order to get access with his broach to the orifice of the root.

At this stage of the proceedings, on motion of Dr. Allport, the following gentlemen, not residents within the geographical limits of the society, were elected as honorary members:

Dr. S. Brown, of New York.
Dr. Perkins, of Rome, New York.
Dr. Smith, of Terre Haute, Indiana.
Dr. Clark, of New Orleans, Louisiana.
Dr. Knapp, of New Orleans, Louisiana.
Dr. Knapp, of Jackson, Mississippi.

EVENING SESSION.

*Subject—Gutta Percha as a base for Teeth.*

Dr. Dunham thinks gutta percha very useful in cases where the teeth have been recently extracted, and the alveolus very prominent, especially about the eye teeth. In these cases it is often very difficult to use metallic plates without making the lips too prominent. He prefers to use a thin plate of gutta percha rather than a gold plate, the ends of the teeth resting on the gum without a plate in front.

Dr. Smith has used gutta percha in two cases, but does not like it; especially the samples which he had, and which were said to have been made by Dr. Slayton. The texture and color were both bad.

Dr. Quinlan is of opinion that gutta percha will be found in many cases to be very useful.

Dr. Spalding uses gold plate, for temporary cases, as the best material. When the gum changes much he uses gutta percha to fill up the interstice or chasm between the plate and the arch of the palate and the alveolar ridge. This plan he finds to work well.

Dr. Clark does not use gutta percha for permanent sets of teeth, but nevertheless regards it as useful for many purposes, particularly when a piece of work constructed upon it brings the patient back in quick time for a new set on proper material. He occasionally uses it for patching up old work, which is generally neither very pleasant nor very profitable to the dentist. He thinks that when a gutta percha plate wears through very soon, it is owing to the material having been overheated, or burned by the lamp.

For lower pieces where only the front teeth are gone, he likes gutta percha, yet he has no reliance upon it for any but temporary sets.

### SECOND DAY.

### *Continuous Gum.*

The Secretary read the minutes of yesterday, which were amended and adopted.

Dr. Clark was requested by Dr. Spalding to state whether solder made with arsenic will eat up the platina plate.

Dr. Clark thinks it will if the arsenic is in excess. The sanguine hopes at first expressed, have not been fully realized. The experiments have not thus far been fully satisfactory, but he hopes the difficulties will be ultimately overcome.

Dr. Honsinger had one job of continuous gum which was not successful, and only one. He proceeded to describe the case minutely. A set of gum teeth had failed in the mouth. He substituted continuous gum. This soon lost one molar, and the gum was broken and loose. Made a new set, and that gave way. Then struck a second plate and added it to the first, bringing the edge over the front enamel. This also gave way, probably on account of the many bakings.

Dr. Clark advised Dr. H. to send his subject to Dr. Harris, of Philadelphia, who once had a similar case, a lower set, which he made of cast-iron.

Dr. Allport said he was gratified to hear gentlemen give us an account of failures, for he thought much more could be learned from some failures than from many successes.

Dr. Spalding said, in relation to the articulation of continuous gum work, it must be good, but when the antagonizing teeth are gone, the danger of unequal pressure can hardly be avoided.

Dr. Perkins had encountered the same difficulty. In such cases he has made the backing and front part of the plate exceedingly strong, and put a block of platina over the place of strongest pressure.

Dr. McKellops has done a good deal of continuous gum work. One piece, after six months, exhibited the four front teeth projecting forward. He split the backings and turned one-half the back up on the plate ; then baked carefully, and merely soldered the backs to the plate, not the pivots.

Had a case where the patient had worn gold plate; first made a block—then substituted gum work. The principal strength in this work is in the baking ; the first bake of the body should be just sufficient to cause the material to adhere.

A young gentleman present was called on by the President to explain his method of making gum work, who described the process generally, but owing to the want of time, too briefly to be of much practical use.

Dr. Blake thinks that the baking should be *progressive*—each baking more than the former, fusing the whole mass.

Described a case of filling up the cheeks, which was fully successful, and is of opinion that continuous gum work is of considerable use to the profession.

Dr. Clark wished to know the experience of the gentlemen present in relation to air chambers. He used them sometimes, not always. He knew of no plainly established rule to govern him when to use them, and when not. In looking back at his practice, he found he had adopted something like the following: When the center of the palatine arch is hard, or has a hard protuberance, or sort of backbone running through its center, he used a chamber, and a large one, but not deep. When the whole arch and gum are spongy, and the membrane thick, he did not use one. He would also like to call attention to the articulating of artificial teeth.

It had been stated that, in natural dentures, a line drawn from the canine to the last molar of the upper jaw, would generally show the teeth to be in the same line.

Artificial teeth cannot have all the uses of natural ones. Natural teeth may interlock by long cusps; artificial ones would be useless articulated thus; and the pieces from our best dentists, and those proving the most useful, are set in a more perfect circle than the natural ones, owing to the necessity of relying mainly on the masseter muscle for their use. Then the lower jaw generally protrudes, so that the circle is necessarily enlarged at the bicuspid part of the arch.

Dr. Dunham is pleased with the character of the discussion this morning. Is obliged to Dr. Honsinger for his particular delineation of his *failures.* This is even more useful to us than a description of our *successes.*

Dr. Dunham sometimes uses air chambers in the cases stated by Dr. Clark, and does not use them in soft spongy gums.

Describes a plan of making air chambers. Cuts a piece of tin an inch long and half or three-quarters of an inch wide; after swaging his plate he places the tin in the female cast and strikes the plate over it. This prevents the rocking.

He takes upper impressions generally in wax; lower ones in plaster.

When the wax is introduced, he directs his patient to press his fingers against the cheek and wax over the molars, whilst he presses up the cup to its position. He presses the front wax a little inward, after it is taken from the mouth.

Dr. Bogue wished the opinion of gentlemen as to air chambers.

Dr. Dunham generally takes three casts, and selects the poorest to swage on at first; then if the fit is bad, on another, and so on to the last if necessary.

Dr. Spalding uses air chambers when the hard prominence exists in the arch. In almost all cases of partial sets he uses air chambers.

Makes the edge of the chamber square and not rounded, to prevent the expansion of muscle into the chamber.

In order to produce the square edge he cuts out a piece of the thin plate. Though shallow, the chamber will not fill with flesh; and its impression is scarcely perceptible to the eye. Considers them better than deep chambers.

On the subject of impressions he has settled upon wax exclusively unmixed.

Uses sheet zinc to strike off a preparatory plate or cup. In this plate he places sheet wax of equal thickness and warmth. When the gum is spongy he cools the wax a little.

In lower plates when the sheet of wax is placed on the zinc plate, he gently presses the wax on the zinc die, to give an *approximation* to the true forms. Takes cold water into the mouth to cool the wax.

In lower plates it was his custom to limit the plate at the movable muscles. Draws a line on his plaster with a pencil where he judges the muscle will move. Increases the suction by remodeling the edge of the plate in front.

One of the most difficult points to fit is behind and around the condyle of the jaw. Attaches the rim to the edge of this curved edge of the plate. This curved edge not only increases the suction, but may be made to fill up the mouth and keep out the lip as desired. Around the condyle it is sometimes necessary to cut out a wedge of the plate. Sometimes injects cold water about the condyle to harden the wax before removing it.

The zinc plate may be one quarter of an inch wider than the gold plate. Does not spread the zinc plate at all at the sides.

Dr. Dunham asks whether Dr. Spalding ever springs the zinc plate, by unequal pressure. The body of wax must be as thin as possible, and not break ; also the wax must be very soft.

A hole must be in the centre of the plate to let in the air before removing.

Sometimes he pares off a little of the plaster cast in front, to compensate for the imperfect fit over the edge of the alveolus.

Dr. Smith, of Ottawa, wished to know whether an *atmospheric pressure plate* is the same as a *suction plate.*

Dr. Spalding thought that capillary attraction had as much to do with the support of the plate in the mouth as atmospheric pressure.

Dr. Dunham differed from Dr. Spalding on the subject of atmospheric pressure ; he believes that atmospheric pressure does exist, because if a small orifice be made into an air chamber the plate will drop.

Dr. McKellops does not know how Dr. Spalding can put a little wax in his plate, and get the soft parts more perfectly than plaster will give. And also how one person finds it necessary to press his wax in in front and around the edge, while Dr. Spalding accomplishes the same thing by carving the wax out at the same points. He must say of central cavities that they are useful. Thinks that in flabby mouths plaster is better than wax.

Dr. Spalding responds that wax impressions caused no more necessary pressure than plaster, if the wax is sufficiently warm.

Engrave a groove in the exact place on the plaster cast where the plate is to terminate on the palate. This forms a flange on the plate for its more perfect adaptation.

Dr. Anderson has thought the air chamber was sometimes used for ornament. Thinks the prettiest form of a plate is that of nature. Thinks the pressure should be removed from the palatal arch for the purpose of avoiding the rocking of the plate. For this purpose he removes an equal surface of the plaster cast, excepting near the posterior part. This plate will ultimately settle about enough to fill up the cavity. Thus the pressure is brought upon the alveolus, where it belongs.

Dr. Clark thinks there need be no misuse of terms in regard to the chambered plates, called atmospheric pressure plates. It seems to him that we have two principles that effect the object, viz. COHESION and ATMOSPHERIC PRESSURE.

Capillary attraction is another thing. It pertains to fluids in connection with tubes, as is shown by the sponge. What is called suction is the *effect* produced by air exhaustion from between two bodies not in contact.

So, then, in the plain plate, we have cohesion (*a well known principle when two bodies are in absolute contact*) and atmospheric pressure.

In the cavity plate we have these two principles and suction, if the air can be exhausted from the chamber. Moisture only assists by lubrication making the contact more perfect, and only assists cohesion, which cannot be called capillary attraction.

Dr. Fitch denies that cohesion or attraction has any thing to do with the plates of teeth. They adhere from atmospheric pressure. Has found air chambers in some cases important.

Dr. Spalding thinks the perfect fit of the margins of the plate more important than the fit in its middle parts.

Dr. Perkins denies cohesive attraction and asserts atmospheric pressure, in the case of plates.

Professor Palmer, of the Michigan Medical University, was requested to explain the terms cohesion and adhesion, and atmospheric pressure.

Dr. Palmer said—Being thus called upon to make remarks in this convention of dentists, though this profession was of a kindred one with his own, was, of course, entirely unexpected; but he had no objection to stating his impressions of some of the points in natural philosophy, which this discussion had suggested.

Attraction is that force or principle in matter by which bodies or their particles are drawn towards each other, or which prevents their separation when united. Aside from chemical and electrical, which are irrelevant to the subject under discussion, writers recognize two kinds of attraction—one operating at *sensible*, and the other at *insensible* distances. The *first* is called attraction of gravitation, and is that which causes a body to fall to the earth, and keeps the whole planetary system in motion and order; the second, attraction of *cohesion*, is that which holds the particles of bodies in contact, giving them consistency, solidity, and strength. This principle of attraction is inherent in all matter—that is, unless opposed by other forces, there is a tendency in matter for its different parts to come together, the tendency increasing with proximity; and when the particles are brought to an insensible distance, they embrace each other by this attractive force—with different degrees of energy, to be sure, depending, in a large measure at least, upon the nearness with which a large number of particles are brought together, causing the more or less solid and tenacious forms of bodies.

When two plane and highly polished surfaces are brought in contact, a large number of particles are brought in the closest proximity, and this cohesive attraction holds them together. When less smooth, but still comparatively hard surfaces are brought in contact, and pressed upon each other with force, with many substances proximity of particles occurs, and cohesion takes place. When certain solid masses are partially liquified or softened as by heat, which softening or liquification is effected by temporarily diminishing cohesive force, more particles are readily brought in contact, and the process of *welding occurs.*

Under certain circumstances, when an unctuous or other semi-fluid substance is introduced, in small quantities, between two surfaces, it serves as a band of union, by the attraction of the material for both surfaces.

*Adhesion* is a general term, signifying the state of sticking to, or being attached by whatever means to something else—*cohesion* being the more scientific or technical term, as applied in a philosophical sense.

Capillary attraction is a modification of cohesive attraction, and is applied to the rising of liquids in small tubes by the attraction between the liquid and the sides of the tubes. The taking up of water by a sponge or cloth is on the same principle—the pores of the sponge and fibres of the cloth acting as the small tubes, and the same term is applied to the process in these cases.

But there is another power which tends to hold two smooth surfaces in contact. This is the pressure of the atmosphere. When surfaces are brought together in such a manner as to exclude the atmosphere, or prevent its introduction at the margins, an attempt to separate these surfaces would tend to produce a vacuum, and would be resisted by this pressure.

Both atmospheric pressure and cohesive attraction operate usually in holding surfaces together—sometimes one and sometimes the other having the greater effect, according to the circumstances of each case.

In keeping plates in the mouth, the atmospheric pressure probably has usually much the more effect. In filling cavities of the teeth with gold, cohesion is the principal effective agent. It is cohesion which causes foil and crystal gold, when pressed firmly together, to form a solid mass, uniting by the same force, as in the case of superficial cavities, the gold to the tooth itself.

The term *suction* indicates the act by which the atmosphere is exhausted from a cavity, and where the atmospheric pressure upon surrounding surfaces forces a fluid or other substance into that cavity.

As applied to a plate in the mouth, the adjective is scarcely as appropriate as other modes of expression.

Adjourned till half-past 2, P. M.

### SECOND DAY—Afternoon Session.

Dr. McKellops moved that the constitution of this Society be published.

Resolved, That the Secretary be requested to furnish Dr. S. Brown with a copy of the constitution for publication in the "Forcep."

The subject of gold as fillings for the teeth, was the order of business.

Dr. Clark said that he had great confidence in gold foil, and thought, if all its properties were understood, it would accomplish much more than was generally expected of it.

He was not prepared to give up his confidence in gold foil, nor was he prepared to say that desirable operations might be performed with crystal gold that could not be done with foil; but some samples of filling which he had accidentally seen of Dr. Allport's of Chicago, led him to suspect such was the case.

One case he would mention. A lady, with two front incissors, which had large approximal cavities, presenting pretty much the appearance of having been separated with a file, a quarter of an inch thick, down two thirds of the distance to the gum. These two teeth were restored perfectly to their original shape. The approximal surfaces, the cutting edges, buccal and lingual surfaces, and all. When he stated the fact that the nerves were both preserved, and that these teeth, bearing the marks of perfect articulation with the lower teeth, had been used for nineteen months, the profession would see the difficulties encountered.

He thought such demonstrations worth all the word pages of theory that could be written on this subject. The class of men who are doing this kind of work encourage us all to make high endeavors in our art.

In thus complimenting Dr. Allport, he does not mean to say that he is going home to throw away his instruments. No such thing, but he is going back to endeavor to imitate h ese great examples.

Dr. Smith once thought he could plug a tooth tolerably well. But he had lately seen a first bicuspid, filled by Dr. Allport, two thirds of which above the gum was of crystal gold. It preserved the original form of the tooth and performed all the purposes of mastication.

Dr. Knapp (of New Orleans) thinks that cylinders are the best form of gold for plugs as a general thing. This system of cylinders used by Dr. Clark, he thinks is new: that any former methods claimed to be such were quite different from this. Crystal gold will doubtless succeed in some cases, especially in building up parts of teeth, such as he had seen of Dr. Allport's, better than any kind of foil.

Dr. Knapp, of Mississippi, was here named and elected an honorary member of the Society.

He returned thanks for the honors, and stated that he has succeeded better with cylinder fillings than with gold in any other form.

Dr. Spalding stated that he had regarded crystal gold as the very best material for filling very shallow cavities; and also in building up structure, the crystal gold is the perfection of material.

Cylinders will make the best filling in the shortest period of time, and with sure labor.

The first specimen of cylinder stoppings I had seen were (ten years ago) by Dr. O. P. Laird, of Columbus, Ga., when he was in Savannah, Ga. He saw also ten years ago, plugs by Dr. Badger, which were very excellent, better than himself can do. He had understood that Dr. Badger used cylinder plugs. He formerly suggested to Dr. Clark the propriety of seeing Dr. Badger—but Dr. Clark has made improvements in the method of forming cylinders. He does not wish to deprive any gentleman of his laurels.

On the whole, he regards cylinder plugs as equal to any other, not excepting crystal gold. The plugs of ordinary form, of crystals and cylinders respectively, are so nearly equal that the difference is not worth regarding.

Dr. Clark had always made it a special aim in all his professional life, to accord to every gentleman all the honors of discovery or introduction of improvements, and he would ever be found punctilious on this point. In the winter of '49, in New Orleans, he formed the acquaintance of Dr. Badger, and became satisfied that Dr. B. could excel him in filling teeth. During a whole season of very friendly intercourse with Dr. B., he never told me how he filled a tooth. He did impart to me my present mode of introducing gold into fangs, which I have given to the profession years ago, in the *Dental Register*; but I refused to receive any thing from him of that nature, that he was not willing the whole profession should know. I never saw him fill a tooth, nor did he ever describe, in the least particular, his mode of using gold. I saw on his table pieces of gold that were in the form of gold wire, and I understood that he filled teeth by using gold in this form, by cutting off such pieces (from this wire, made from rolled foil) as he needed, of the length required, according to the depth of the cavity.

I commenced preparing my gold by rolling gold of the length wanted, so that I need not harden the ends by the cutting. I soon caught the principle of cohesion of surfaces in contact, and established my present mode of cylinder filling; and after a year or two found out that Dr. Badger did not fill with cylinders, and that he asserted so himself. That he packed his gold around the walls, and thrust these sections of cylinders into the centre. Of my whole course in this direction I have much to cheer me, and nothing to explain or regret. But I will say, that it glads me more, that my professional brethren have found this useful, and of practical service in their operations, than anything of a congratulatory nature.

Dr. Allport thinks the profession are pretty well aware who is entitled to the credit of inventing this method of filling teeth, and whoever he is, he enjoys the satisfaction of knowing it.

Dr. Knapp (younger) says there is nothing before this body to show that Dr. Laird, ten years ago, filled teeth with cylinders.

Dr. Kennicott says that, as long ago as 1820, he saw Dr. Cox, in New Orleans, fill teeth with cylinders.

He has failed with crystal gold, many times. Not a particle of moisture must touch a gold filling of any kind.

Dr. Honsinger thinks that crystal gold has mostly failed in consequence of having been introduced in too large quantities. He uses very small instruments. Has built up teeth when one third gone.

Dr. Bogue gives his testimony decidedly in favor of crystal gold.

Dr. Clark has used but little crystal gold, but wishes to know whether the serrated instruments do not fracture the surface of the cavity, in consequence of their sharpness.

Dr. Perkins—I have some experience in this department of filling with foil. If any persons will come to Rome, I will show them fillings built up with foil by Dr. Allport, eight years ago, in fine condition now.

I have used Watts' gold from the commencement of his manufacture, and regard it a very useful adjunct to foil. Has recently used about one third crystal gold.

Does not think a person ought to say that foil cannot be made to perform any thing that crystal gold can do. Nor can he assert positively that it can.

He described at length the manner of stopping with crystal gold.

Dr. Clark requested that Dr. Allport would describe his method of stopping with crystal gold.

Dr. Allport could hardly express his gratitude for the kind and flattering compliments which had been bestowed upon him.

Does not use crystal gold exclusively. Foil constitutes about two thirds of his fillings. Sometimes he uses pellets and cylinders and crystals all in the same cavity. The use of crystal gold requires four times the time for insertion that is required by foil.

Never saves any more of the enamel than is strong and healthy—but saves all the front possible. He presses the crystals in the direction of the length of the tooth as much as possible. Uses serrated instruments, small and sharp.

If a plug gets wet when of foil or pellets, or crystals, it can be cleaned by chloroform and wiped dry with bibulous paper.

The credit due to crystal gold is attributable to Dr. Watts. Since July last his gold has been good, very good—all that is required.

He can build upon a surface after it has been polished, or upon solid metal.

Dr. Spalding explained the reason why serrated instruments packed the crystals in such a manner as to cause them to cohere in a mass, but it must be borne in mind that the serrated points do not push all parts of the crystals with equal solidity; at the polished surface they become nearly so.

Dr. Dunham does not think he could stop front teeth better with crystals than with foil; he never allows the force to fall wholly upon one tooth, but introduces a wedge between the teeth, to connect the force with two or more teeth.

The society next proceeded to the consideration of

*Continuous Gum Work.*

Dr. Spalding thinks the gum and body may be made strong enough.

Dr. Kennicott gives his word for continuous gum work, and esteems it the perfection of artificial teeth.

Dr. Clark is using it pretty extensively, and thinks it occupies an important place in the profession. Deems it as useful as any kind of work that has ever been made. It is artistic in an eminent degree, and is not liable to the objections raised against gutta percha, that it lowers the standard of professional skill.

Dr. Spalding assents to all that Dr. Clark has said in favor of continuous gum, yet there is one serious objection, which is the liability of breaking the teeth either in the process of manufacture, or by a fall afterwards, in which case it is a sort of work very difficult to repair.

Dr. Dunham regards the breaking of the gum as a much greater objection than the breaking of the teeth. This he has not been able to avoid.

Dr. Kennicott has seen gum work that nothing else has ever equalled. It can be adapted in color both of gum and teeth to suit the complexion. The frequent heating of this work is very liable to injure the teeth.

Dr. Perkins regarded it as unfit in all cases.

Dr. Smith desired to know whether tobacco did not make gutta percha sets very disagreeable. Dr. Clark replied that a smoker would find his teeth disagreeable.

Dr. Dunham has charged $75 for sets of gutta percha teeth, which is the regular price in St. Louis. Thinks twenty-five of his patients are wearing them comfortably.

Dr. Allport has used it in cases where it answered a very good purpose for temporary work.

Dr. McKellops moved that when this Society adjourn it be to meet in the city of St. Louis, on the third Monday in May next. Adjourned.

In the evening of the 31st an entertainment was given by the dentists of Chicago, at the Briggs' Hotel. In addition to the many entertaining and instructive speeches by the members of the dental profession, Dr. Palmer, one of the professors of the Michigan Medical University, who knows exactly what to say, with decided ability to say what he thinks and wishes in the most fluent and condensed phraseology, made two eloquent addresses, with which the members of the Society and invited guests were greatly delighted.

Dr. Brown gave as a sentiment—" The Medical Profession —the primitive stock from which ours arose."

Dr. A. B. Palmer, Professor in the Medical Department of the University of Michigan, one of the editors of the Peninsular Journal of Medicine, &c., &c., being present as an invited guest, was called upon, and in response said, in substance—That though not entirely unaccustomed to speaking to numbers, he *was* unaccustomed to after-dinner speeches, and especially did he find it embarrassing to attempt to make a speech, after such extensive and efficient use as had just been made of his dental apparatus, repaired and made effective as it had been by the very skillful member of this body sitting at the head of the table. He could not, however, refuse to respond to the sentiment just offered to the profession which he loved, and to which he had given, so far, the best energies of his active life.

He was happy in being present on this occasion, and of the opportunity of mingling in sentiment and feeling with the members of the Western Dental Convention. The professions of medicine and dentistry were kindred. They had been compared to a parent plant and its offshoot, and the comparison was just.

The two professions were similar in several respects. They were kindred in requiring alike an acquaintance with several branches of knowledge—of anatomy, physiology, pathology, and therapeutics; particularly so far as these have relations to the important organs which come especially under the care of those of your fraternity; and if it be regarded as within your province to consider the causes which lead to the decay of the teeth, you are taken over a large range in common with us. Indeed, the health of the general system is essential to the perfection of the teeth, and upon the perfection of these organs, or at least, their freedom from disease, depends in no small degree the health of the system. Thus have we many subjects of study in common, and thus are our professions kindred. It is true that the medical profession has a wider range of subjects of study and action—we have more to do with the profound mysteries of life; but as ours has more to do with the secret and hidden, yours is more engaged with the open and apparent, and is, consequently, more positive and certain.

But in another, a higher and a better sense, are our professions kindred. We are alike engaged in the relief of suffering humanity—we are engaged in arresting and repairing the ravages of disease and accident in the human frame—we are engaged in the God-like business of doing good. Healing the sick—restoring the maimed, was no inconsiderable portion of the mission of the Divine Master. This is our mutual occupation, and this should make both of our professions high and noble—should make them liberal and benevolent *professions*, and not mercenary trades.

The advantages of associations like the present—of the bringing together a body of men such as these from distant localities, for the purpose of promoting a common and laudable pursuit, are many and great. There is a free interchange of ideas—the particular modes of practice peculiar to each are freely presented for the improvement of all. As was said by Dr. Clark, many things were received, as it were, by absorption—many ideas are unconsciously transposed from one to another by the contact. This reception of ideas by personal intercourse is more effective than by reading. The personal presence of the individual impresses more strongly the fact or the principle he would communicate, and, besides this, each would feel a greater interest in what another writes, from having seen and known him.

But the direct increase in knowledge and familiarity with the different modes of practice, are not the only or even the principal advantages. A spirit of improvement is awakened—a desire for advancement is excited—and thus would a higher position be attained. And there was need of inducements of this nature. Popular judgment of professional merit is notoriously erroneous—the noisy pretender often enjoys a larger share of public favor than the more quiet man of merit. A more correct appreciation exists among one's professional brethren. They alone are competent accurately to judge, and of a reputation among those of his own profession, one may justly be proud. These associations tend to establish such reputations, and to induce real improvement for the sake of them. And these advantages are not entirely confined to those who may be present on such occasions, but the impulse will be felt throughout the whole profession. The absent must arouse themselves, or they will be left behind in the march of improvement.

But there were other, and what might be regarded as incidental advantages of these meetings. They afforded the most agreeable social occasions. There was great pleasure in the extension of agreeable acquaintances, and the union of the States was strengthened by uniting men in a common cause, and bringing them around the same hospitable board, all the way from Wisconsin to Louisiana; and the time might come when all such aids would be needed.

He could not sit down without expressing his gratification at the repudiation he had heard from those present of secrets in their profession. In this respect it should be placed on the same footing with the medical profession. With medical men, no one could maintain a respectable position who held a secret remedy or a secret process; and dentists owed it to themselves, as members of a liberal profession, to exclude from their communion and fellowship all those who were unwilling that their brethren and suffering humanity should have the full benefit of their discoveries and improvements. On this subject he could not express himself more strongly than he felt. On no other grounds could he recognize the profession of dentistry as kindred with that of medicine.

In conclusion he would give—"The Dental Profession, as distinguished from a mercenary trade."

The Chicago meeting of the "Western Dental Society" will long be remembered, with pleasure and profit, by those present.

